# Heart failure with preserved ejection fraction: implications for anaesthesia

**DOI:** 10.1016/j.bjae.2024.02.003

**Published:** 2024-03-06

**Authors:** A. Shah, N. Sabharwal, J.R. Day

**Affiliations:** 1 Nuffield Division of Clinical Neurosciences, University of Oxford, Oxford, UK; 2 Oxford University Hospitals NHS Foundation Trust, Oxford, UK

**Keywords:** diabetic ketoacidosis, heart failure, HFpEF, SGLT2 inhibitors

**Learning objectives**

By reading this article, you should be able to:

• Describe the epidemiology and pathophysiology of heart failure with preserved ejection fraction (HFpEF).
• Discuss the diagnostic work-up of patients in whom a diagnosis of HFpEF is suspected.
• Outline the pharmacological and non-pharmacological management of HFpEF.
• Summarise the perioperative considerations in patients with HFpEF undergoing major surgery.
• Safely manage patients taking sodium-glucose cotransporter type 2 (SGLT2) inhibitors in the perioperative period.

**Key points**

• The numbers of patients with HFpEF presenting for surgery are likely to increase.
• Diagnosing HFpEF relies on the presence of clinical signs and symptoms, and echocardiographic evidence of preserved left ventricular (LV) systolic function and increased LV filling pressures.
• First-line management of HFpEF includes sodium-glucose cotransporter type 2 (SGLT2) inhibitors, diuretics and treatment of coexisting comorbidities such as hypertension and diabetes. Atrial fibrillation should be managed aggressively.
• SGLT2 inhibitors should be stopped 2–3 days before elective surgery to minimise the risk of developing euglycaemic diabetic ketoacidosis in the perioperative period.

Globally, >60 million people are estimated to have heart failure (HF), of which heart failure with preserved ejection fraction (HFpEF) accounts for 50% of cases.^1^ The prevalence of HFpEF is steadily increasing because of improved recognition, an ageing population and reduction in death from comorbidities that can lead to HFpEF. This review provides an update on the epidemiology, pathophysiology, diagnosis and management and perioperative considerations for patients with HFpEF.

## Definitions

**Fig 1:**
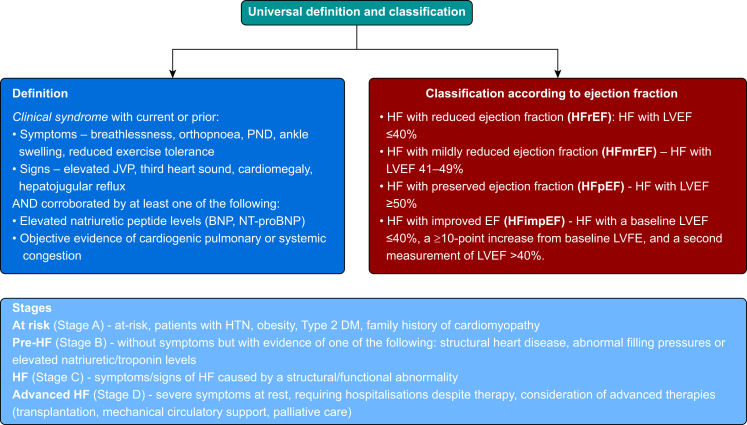
Universal definition and classification system for heart failure. DM, diabetes mellitus; HTN, hypertension; JVP, jugular venous pressure; PND, paroxysmal noctural dyspnoea.

Previous definitions of HF were largely arbitrary and lacked standardisation. In 2021, various cardiology societies developed a universal definition and classification system for HF (Fig. 1) designed to be clinically relevant with prognostic and therapeutic validity, applicable globally and allow standardisation of endpoints in research.^2^ The definition describes HF as a clinical syndrome with current or prior:

(i) Symptoms, signs, or both caused by a structural or functional cardiac abnormality
(ii) Corroborated by at least one of the following: (a) increased natriuretic peptide concentrations, (b) objective evidence of cardiogenic pulmonary or systemic congestion through imaging (echocardiography) or haemodynamic measurement (e.g. right heart catheterisation) at rest or exercise.

Other syndromes may also fulfil this definition of HF. Examples include right HF (the commonest cause of which is left HF), cardiogenic shock, acute coronary syndrome, congenital heart disease, valvular heart disease, high-output failure and hypertensive crises. Management should be guided towards treating the underlying cause and treating the symptoms of HF.

## Epidemiology

Current global patterns demonstrate that whilst the incidence of HF has stabilised and is possibly declining in industrialised nations, the prevalence is increasing because of an ageing population and improvements in medical care.^1^ In a UK population-based study of 4 million individuals between 2002 and 2014, the incidence of HF decreased by 7% (from 358 to 332 per 100,000 person-years) but the absolute number of people diagnosed with HF increased by 12%.^3^ The prevalence remained stable between 1.5% and 1.6% but the absolute number increased by 23%. Socioeconomically deprived people were more likely to develop HF compared with affluent people and did so earlier in life.^3^ These data are consistent with a meta-analysis of >6 million individuals which found that low socioeconomic status was associated with an increased risk of incident HF ranging between 43% and 87%.^4^

## Pathophysiology

Heart failure with preserved ejection fraction now accounts for 50% of cases of HF.^5^ One large, multicohort study demonstrated an increase in the incidence of HFpEF from 4.7 to 6.8 per 1000 person-years over two decades.^6^ Risk factors for developing HFpEF include increasing age, hypertension, obesity, diabetes and previous myocardial infarction.^7^

Several pathophysiological pathways have been proposed, and a detailed review can be found elsewhere.^8^^,^^9^ The historical model is one of hypertension with a hypertensive heart developing diastolic dysfunction (DD) with systolic dysfunction then developing over time. Treatment of hypertension has been shown to reduce incidence of HFpEF by 40% over 2–8 yrs.^10^ Differences exists in the pathophysiology of left ventricular (LV) function between HFpEF and HF with reduced ejection fraction (HFrEF). Heart failure with preserved ejection fraction is associated with impaired ventricular relaxation, increased stiffness and increased filling pressures with pressure overload, whereas in HFrEF, the left ventricle undergoes eccentric remodelling resulting in chamber dilatation with volume overload.^5^^,^^8^

Recent data suggest a proinflammatory state induced by comorbidities such as obesity, diabetes, chronic obstructive pulmonary disease (COPD) and chronic kidney disease which cause systemic microvascular endothelial inflammation with downstream myocardial inflammation, increased oxidative stress and deregulation of nitric oxide signalling, fibrosis and hypertrophy.^5^^,^^11^ Interleukin (IL)-6 concentrations are commonly increased in HFpEF and are associated with greater symptom severity, poor exercise capacity and increased upper body fat composition.^12^

## Diagnosis

Current European Society of Cardiology guidelines recommend a simplified, pragmatic approach which should include the following:^13^

(i) The presence of symptoms and signs of HF
(ii) LV ejection fraction (LVEF) ≥50%
(iii) Exclusion of other pathologies that can mimic HFpEF
(iv) Evidence of increased LV filling pressures/DD/increased left atrial volume and raised natriuretic peptides

Approximately two thirds of patients with HFpEF present with dyspnoea and clinical signs of congestion such as peripheral oedema, raised jugular venous pressure and ascites.^5^ Those with unexplained dyspnoea without evidence of congestion require further diagnostic testing. As outlined above, patients diagnosed with HFpEF often have other comorbidities such as pulmonary disease, anaemia and atrial fibrillation which should be investigated and managed accordingly.^14^^,^^15^

Differential diagnoses of cardiac origin can be split into those affecting the myocardium and those that affect loading conditions. Conditions affecting the myocardium include coronary artery disease, either epicardial or microvascular. Infiltrative cardiomyopathies include amyloidosis, sarcoidosis, hypertrophic and storage diseases such as haemochromatosis. Loading conditions include hypertensive disease, left-sided valvular disease, pericardial disease, arrhythmias and conditions leading to a high-output state.

**Fig 2:**
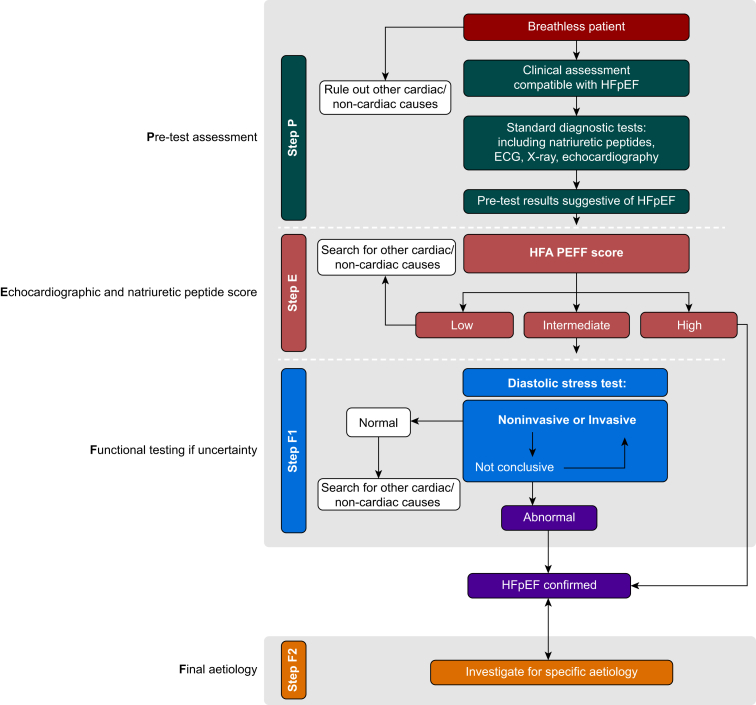
Suggested diagnostic algorithm for HFpEF in patients with unexplained dyspnoea.

Differential diagnoses for HFpEF include hypertrophic cardiomyopathy, amyloid cardiomyopathy, pulmonary hypertension, constrictive pericarditis and coronary artery disease.^5^ In cases of diagnostic uncertainty or unexplained dyspnoea, various algorithms have been proposed. Pieske and colleagues propose a stepwise HFA-PEFF algorithm which uses four steps (Fig. 2).^16^ The H2PEF score is also recommended by guidelines to estimate likelihood of HFpEF in unexplained dyspnoea (Table 1).^17^^,^^18^ In areas of high disease prevalence, a score of ≥6 is associated with >95% probability of HFpEF whereas a score of 0–1 was associated with <25% probability of having HFpEF.^17^

**Table 1:** H2PEF score to assess risk of HFpEF in unexplained dyspnoea.

Clinical variable	Points
**H**eavy (BMI >30 kg m^−2^)	2
**H**ypertensive (>2 antihypertensive medications)	1
**F**ibrillation, atrial (any history)	3
**P**ulmonary hypertension (rest RVSP >35 mmHg)	1
**E**lderly (age >60 yrs)	1
**F**illing pressure (E/e’>9)	1

Score	0–1	HFpEF ruled out
	2–5	Consider rest/stress RHC or stress echo
	6–9	HFpEF ruled in

BMI, body mass index; E/e′, ratio of early diastolic mitral inflow blood velocity to mitral annular tissue velocity; RHC, right heart catheterisation; RVSP, right ventricular systolic pressure.

Brain natriuretic peptide (BNP) and N-terminal fragment of the prohormone BNP (NT-proBNP) are produced by cleavage of the proBNP, a precursor molecule, in normal cardiomyocytes. Their release is triggered by high LV end-diastolic wall stress, which is inversely proportional to wall thickness. Multiple studies have demonstrated that NT-proBNP concentrations have a high negative predictive value (94–99%) for excluding HF in acute (BNP <100 pg ml^−1^ or NT-proBNP <300 pg ml^−1^) and non-acute (BNP <35 pg ml^−1^ or NT-proBNP <125 pg ml^−1^) settings.^13^

However, in HFpEF, LVH can normalise wall stress and therefore natriuretic peptide concentrations can be falsely negative. Up to 20% of patients with HFpEF diagnosed by invasive testing have concentrations below the recognised diagnostic thresholds.^16^ Factors associated with normal BNP concentrations (<100 pg ml^−1^) in patients with HFpEF are younger age, female sex, obesity, presence of sinus rhythm, normal renal function, or both and caution is advised in using these peptides to rule out HFpEF in these groups.^5^^,^^19^

## Diastolic dysfunction

Diastolic dysfunction is an important aspect of HFpEF but does not account for the entirety of its pathophysiology. It should be noted that diastolic function deteriorates as a natural part of ageing. Left ventricular DD refers to a disorder of ventricular relaxation and an increase in chamber stiffness. Risk factors for developing DD include hypertension, coronary artery disease, increasing age, female sex and renal impairment.^20^ As DD progresses, it can become unmasked during periods of stress where myocardial function is stressed beyond its physiological reserve (e.g. fluid overload, severe hypertension, atrial fibrillation). Typical signs and symptoms include those of congestion.

Guidance on diagnosing DD is available from the American Society of Echocardiography.^21^

Important basic clinical considerations when assessing for DD are heart rate and rhythm, blood pressure and fluid status. The echocardiographic features should be assessed in relation to LV size, volume, wall thickness and ejection fraction. An assessment of left atrial volume and mitral valve function should also be included.

The variables that are key to assessing DD are mostly measured in the apical four-chamber view and include the mitral inflow Doppler flow measured at the tips of the mitral valve, tissue Doppler (TDI) at the medial and lateral mitral annulus. Other variables include left atrial volume and size and the velocity of a tricuspid valve regurgitant jet. The presence or absence of DD is based on the following four variables and their cut-off values:

(i) Septal e′ <7 cm s^−1^ or lateral e′ <10 cm s^−1^
(ii) Average E/e′ ratio >14
(iii) LA volume index >34 ml m^−2^
(iv) Peak TR velocity >2.8 m s^−1^

Diastolic dysfunction is present if more than half of the available variables are abnormal.

## Treatment

First-line management of HFpEF includes sodium-glucose cotransporter type 2 (SGLT2) inhibitors, diuretics for fluid retention and treatment of other cardiovascular and non-cardiovascular comorbidities. All are Class 1 (evidence, general agreement, or both that a given treatment/procedure is beneficial, useful, effective, or all) recommendations from the updated ESC 2023 guidelines.^22^

### SGLT2 inhibitors

Sodium-glucose cotransporter type 2 inhibitors are first-line therapy for diabetes mellitus and act by inhibiting the SGLT2 protein, which inhibits reabsorption of glucose in the kidney which in turn lowers blood glucose. Examples include empagliflozin and dapagliflozin; a dose of 10 mg once daily is recommended in HF. Several mechanisms of action of the cardioprotective effects of SLTG2 inhibitors have been proposed. Sodium-glucose cotransporter type 2 inhibitors increase circulating ketone concentrations, which provide an additional fuel source for the failing heart, thus improving cardiac energetics and efficiency.^23^^,^^24^ These drugs also have anti-inflammatory properties and animal models have demonstrated antifibrotic effects after myocardial infarction.^25^ Other important beneficial effects include diuresis, improved blood pressure control, erythropoiesis, reduction in hyperuricaemia and decreased oxidative stress—a detailed review of these can be found elsewhere.^26^

Meta-analysis of pooled data from five randomised controlled trials (RCTs), involving 21,947 patients, found that SGLT2 inhibitors reduced cardiovascular-related death after hospitalisation for HF (relative risk [RR] 0.77, 95% confidence interval [CI]: 0.72–0.82), cardiovascular death (RR 0.87, 95% CI: 0.79–0.95), first hospitalisation for HF (RR 0.72, 95% CI: 0.67–0.78) and all-cause mortality (RR 0.92, 95% CI: 0.86–0.99).^27^ These effects for each of these outcomes were also consistently observed in patients with HFpEF or HFrEF.

**Fig 3:**
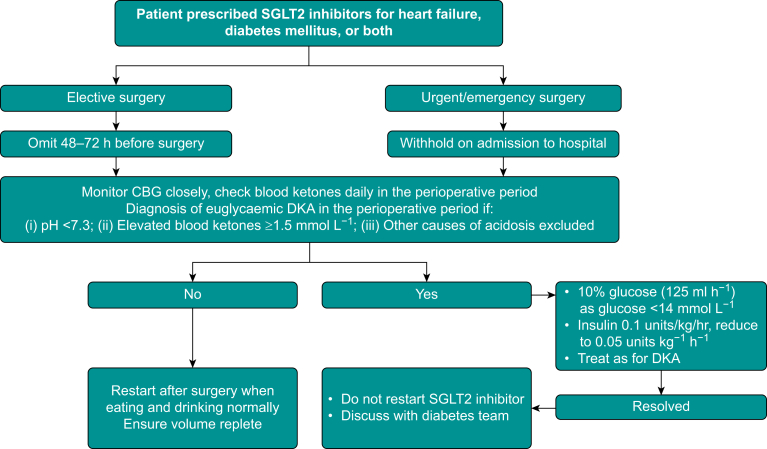
Perioperative management of SGLT2 inhibitors and suspected euglycaemic DKA. CBG, capillary blood glucose.

Sodium-glucose cotransporter type 2 inhibitors are contraindicated in patients with a history of diabetic ketoacidosis (DKA), type 1 diabetes, recurrent genitourinary infections or estimated glomerular filtrate rate (eGFR) rate <20 ml min^−1^ 1.73 m^−2^. Small increases in creatinine and glycosuria are expected. However, SGLT2 inhibitors are well tolerated with pooled data demonstrating no increased risk of adverse events (hypoglycaemia, renal adverse events, amputation, DKA) when compared with placebo.^28^ Perioperative management of SGLT2 inhibitors is outlined in Figure 3.

### Diuretics

Loop diuretics, such as furosemide, torsemide, or bumetanide, are first-line therapy in patients with clinical features of congestion. Torsemide and bumetanide have higher bioavailability than furosemide but current evidence does not demonstrate superiority of one agent over another.^29^

### Neurohumoral antagonists

Early studies showed that established therapies used for HFrEF, such as mineralocorticoid receptor antagonists (MRAs), angiotensin converting enzyme (ACE) inhibitors, angiotensin receptor blockers (ARBs), angiotensin receptor–neprilysin inhibitors (ARNIs), largely yielded neutral results in patients with HFpEF.^30^ However more recent data support the addition of these therapies in patients who are already established on SGLT2 inhibitors, and the following may be considered:^5^

• MRAs (e.g. spironolactone)—if EF <60%, increased BNP, eGFR >30 ml min^−1^ 1.73 m^−2^, normal serum potassium, recent HR admission
• ARNIs (e.g. sacubitril valsartan)—if EF <45% for men/EF <60% for women, increased BNP, risk factors for HF admission
• ARBs (e.g. candesartan)—if EF <55%

### Glucagon-like 1 peptide receptor agonists

Glucagon-like 1 peptide (GLP-1) agonists (e.g. semaglutide) are recommended for the management of type 2 diabetes and obesity. Their mechanisms of action include promoting glucose-independent insulin secretion, delaying gastric emptying and inhibiting postprandial glucagon secretion. Given the high prevalence of these comorbidities in patients with HFpEF, pharmacological reduction of weight could be beneficial. A recent randomised trial evaluated the effect of once weekly semaglutide (2.4 mg) injections or placebo in patients with HFpEF and BMI ≥30 kg m^−2^, and found that semaglutide treatment was associated with fewer HF-related symptoms, greater weight loss and improvement in exercise capacity.^31^

### Non-pharmacological strategies

Aerobic exercise training, cardiac rehabilitation and diet-induced weight loss have been shown to lead to meaningful improvements in exercise performance and quality of life.^32^, ^33^, ^34^ Patients with HFpEF have worse physical impairments than those with HFrEF at baseline but may also derive the greatest benefit from cardiac rehabilitation.^33^ For patients with HFpEF and obesity, calorie restriction through prepared meals to achieve reduction in energy intake by 400 kcal per day can result in modest weight loss and improvement functional performance (change in peak oxygen consumption of 1.3 ml kg^−1^ min^−1^).^34^

### Pacing

Chronotropic incompetence (inability to reach expected peak heart rate) is common in patients with HFpEF and associated with poor exercise tolerance. Two studies have evaluated the role of improving heart rate response with pacing on exercise performance, albeit with conflicting results. In the Rate-Adaptive Atrial Pacing in Diastolic Heart Failure (RAPID-HF) RCT, insertion of a pacemaker to increase heart rate did not improve exercise capacity and was associated with increased adverse events.^35^ However, the study was single centre, enrolled only 29 participants and faced several barriers including high participant burden, lack of equipoise from many cardiologists and a lengthy enrolment period (2014–22). In contrast, the myPACE RCT enrolled 106 patients with HFpEF and pre-existing pacemakers for ventricular conduction and randomly allocated them to either a personalised backup heart rate setting (myPACE algorithm) or usual care (pacemaker backup rate remained at 60 beats min^−1^).^36^ Treatment with the myPACE algorithm was associated with improvements in quality of life, NT-proBNP concentrations, physical activity and atrial fibrillation. The ongoing PACE HFpEF study is investigating the role of continuous heart rate elevation (with and without superimposed nocturnal pacing with a dual chamber pacemaker targeting the Bachmann bundle and the bundle of His) on quality of life, NT-proBNP concentrations and 6-min walk distance in patients with HFpEF. At present, the role of cardiac pacing in patients with HFpEF remains unclear.

## Perioperative considerations in patients with HFpEF

Patients with HFpEF experience high rates of postoperative morbidity and mortality after non-cardiac surgery. In one database study involving 153,771 patients with HFpEF, any cardiopulmonary complications (e.g. acute coronary syndrome, respiratory failure) occurred in 40.8% and non-cardiopulmonary complications (e.g. renal failure, sepsis, stroke) in 54.5%. In-hospital mortality was 4.6% and 30-day readmission was 20.2%.^37^

### Preoperative

Functional capacity should be assessed using subjective and objective methods. Preoperative cardiology consultation and optimisation is recommended. Patients with HFpEF often have other comorbidities (Table 2) and these should be optimised where possible. An important consideration is the management of SGLT2 inhibitors (Fig. 3).

**Table 2:** Management of coexisting comorbidities in patients with HFpEF.

Condition	Recommendation
Hypertension	Manage according to local/national guidelines and aim SBP <130 mmHg
Coronary artery disease	Antiplatelet therapy Consider revascularisation in patients with inducible ischaemia Statin therapy if increased LDL
Atrial fibrillation	Anticoagulation for stroke prophylaxis Consider rhythm control
Diabetes	Optimise diabetes control (prioritise SGLT2 inhibitors) and manage micro/macrovascular complications accordingly
Chronic kidney disease	ACE inhibitor/ARB for proteinuria SGLT2 inhibitors
Obesity	Calorie restriction and aerobic exercise training Consider GLP-1 receptor agonists Screening and treatment for OSA
Anaemia	Screen and treat for iron deficiency

ACE, angiotensin converting enzyme; ARB, angiotensin receptor blocker; GLP, glucagon-like peptide; LDL, low density lipoprotein; OSA, obstructive sleep apnoea; SBP, systolic blood pressure; SGLT2, sodium-glucose cotransporter type 2.

### Intraoperative

The goals of anaesthesia are to understand and predict the likely changes and reactions to stages in the perioperative process. Major distrubances in physiology tend to occur with induction of anaesthesia, major bleeding, inflammatory responses associated with sepsis and the change from invasive to spontaneous ventilation and extubation.

In patients with HFpEF there is loss of relaxation of the heart to aid filling in diastole. Tachycardia is poorly tolerated in patients with HFpEF, because of shortened diastolic filling time. An increase in left atrial pressure is transmitted to the pulmonary veins and the pulmonary capillaries, potentially leading to increased transduction of fluid into the alveolar spaces.

Atrial fibrillation is important and can be problematic for two reasons. It has a tendency to cause tachycardia but there is also the loss of coordinated atrial contraction. In HFpEF the atrial contraction is correspondingly more important in aiding ventricular filling during diastole. In physically fit young subjects, ventricular filling occurs almost completely during early diastole with little contribution from the atrial contraction or ‘kick’. This can be seen in echocardiography where the A wave is almost non-existent as there is no mitral inflow because the ventricle has been almost filled by the early filling wave or E wave.

Patients with HFpEF are much more sensitive to dynamic changes in their volume status. This is often referred as having ‘teaspoon physiology’. They require an adequate driving pressure to ensure adequate diastolic filling with a high left atrial pressure but lack compliance so can easily and rapidly have an escalating increase in left atrial pressure if the volume status rapidly rises. Putative mechanisms include higher sympathetic nervous system tone and altered beta adrenoceptor sensitivity. Loss of the sympathetic tone with general or neuraxial anaesthesia can lead to exaggerated hypotension.

The use of chronic medications that affect vascular tone and fluid status such as vasodilating antihypertensive drugs (e.g. ACEIs, ARBs and diuretics) can exacerbate these changes. General anaesthetic agents can also have a direct effect on cardiac contractility and lusitropy. Anaesthesia should be induced in a smooth and controlled manner. Direct arterial pressure monitoring and arterial waveform analysis may be needed, depending on the context of the surgery. The dose of induction agent should be reduced and onset of anaesthesia may be delayed because of a prolonged circulation time.

There are few data on the effects of common anaesthetic agents on diastolic function. A study assessing the effect of isoflurane, sevoflurane and desflurane showed no change in diastolic function both in healthy volunteers and those with DD. Sarcolemmal transport of calcium ions is reduced by the intravenous anaesthetic agents barbiturates and ketamine. This can lead to a reduction in chamber compliance. Diastolic performance seems to be unaffected by agents such as etomidate, propofol, morphine, midazolam and remifentanil. Close maintenance of oxygen and carbon dioxide partial pressures are recommended to avoid acute changes in pulmonary pressure.

### Postoperative care

On emergence of anaesthesia there is often an acute increase in sympathetic tone, which can precipitate hypoxaemia and tachyarrhythmias including atrial fibrillation. There may also be a rapid move of fluid to the central vasculature particularly with the resolution of any neuraxial block. The non-compliant heart may tolerate this fluid shift poorly and could precipitate acute HF.

Close vigilance for euglycaemic diabetic ketoacidosis (eDKA) is recommended (Fig. 3). Euglycaemic diabetic ketoacidosis is likely to be underdiagnosed because of its atypical presentation. Although this is most likely to occur in patients with diabetes mellitus, isolated cases in patients without underlying diabetes have been reported.^38^ Reported risk factors include dehydration, concurrent acute illness, degree of surgical stress, changes to diabetes medications (particularly insulin being reduced or withheld) and reduced carbohydrate intake.^39^ Sodium-glucose cotransporter type 2 inhibitors can be restarted after surgery as soon as patients are able to eat and drink normally and are euvolaemic. In those who develop eDKA, SGLT2 inhibitors should be discontinued and only restarted, if indicated, by the diabetes team.

## Overall prognosis of HFpEF and directions for future research

Heart failure with preserved ejection fraction is associated with poor quality of life and worse clinical outcomes when compared with patients with HFrEF.^5^^,^^33^ In-hospital mortality ranges from 2.4% to 5% and 1-yr mortality varies from 20% to 29%.^40^ After a hospital admission for HFpEF, 5-yr readmission rates are 80% and mortality rates range from 35% to 75%—worse than many cancers.^40^ Cardiovascular causes are the primary cause of death in HFpEF and non-cardiovascular causes of death comprise a higher proportion of deaths in HFpEF compared with HFrEF.^41^

Although significant advances into the epidemiology, pathophysiology and management of HFpEF have been made over the past decade, significant knowledge gaps remain. Heart failure with preserved ejection fraction is heterogeneous and identifying distinct phenotypes for predictive and prognostic enrichment is an active area of research.^9^ Large-scale studies using electronic health records may help in this area.^42^ Recent data also support the hypothesis that HFpEF is a pro-inflammatory state and further research is needed into the efficacy and safety of anti-inflammatories (e.g. IL-6 antagonists) in HFpEF. Most current epidemiology data are from Europe and North America, and data from other populations are needed including from low-middle income countries.

## Declaration of interests

The authors declare that they have no conflicts of interest.

## Acknowledgements

We would like to thank Dr Shrinivas Sharma (consultant endocrinologist and diabetologist, Great Western Hospitals NHS Foundation Trust) and Dr Alistair Lumb (consultant endocrinologist and diabetologist, Oxford University Hospitals NHS Foundation Trust) for the input regarding the perioperative management of SGLT2 inhibitors and eDKA.

## MCQs

The associated MCQs (to support CME/CPD activity) will be accessible at www.bjaed.org/cme/home by subscribers to *BJA Education*.
